# Clinical significance of C-Reactive Protein to Lymphocyte Count Ratio as a prognostic factor for Survival in Non-small Cell Lung Cancer Patients undergoing Curative Surgical Resection

**DOI:** 10.7150/jca.58094

**Published:** 2021-05-27

**Authors:** Jae-Joon Hwang, Joon Young Hur, Wankyu Eo, Soomin An, Dae Hyun Kim, Sookyung Lee

**Affiliations:** 1 Department of Allergy, Pulmonary and Critical Care Medicine, Gachon University Gil Hospital, Incheon, Republic of Korea.; 2 Department of Internal Medicine, Hanyang University Guri Hospital, Hanyang University College of Medicine, Guri, Republic of Korea.; 3 Department of Medical Oncology & Hematology, College of Medicine, Kyung Hee University, Seoul, Republic of Korea.; 4 College of Nursing, Hallym Polytechnic University, Gangwon-do, Republic of Korea.; 5 Department of Thoracic Surgery, Kyung Hee University Hospital at Gangdong, Seoul, Republic of Korea.; 6 Department of Clinical Oncology, College of Korean Medicine, Kyung Hee University, Seoul, Republic of Korea.

**Keywords:** Carcinoma, Non-Small Cell Lung, Pulmonary Surgical Procedures, Prognosis, Lymphocyte Count, C-Reactive Protein

## Abstract

**Purpose:** We assessed the clinical feasibility of C-reactive protein to lymphocyte ratio (CLR) as a determinant of survival in patients with non-small cell lung cancer (NSCLC) undergoing curative surgical resection.

**Methods:** A retrospective study was conducted on patients with stage I and II NSCLC undergoing curative resection. Demographic and clinical variables, including CLR, were collected and analyzed. The Cox proportional hazards model was used to calculate hazard ratios for overall survival (OS) and cancer-specific survival (CSS). The Mann-Whitney U test was used to compare differences between two independent groups.

**Results:** The median age of the patients was 69.0 years, and male patients comprised 63.9% of all patients. A total of 164 (75.9%) patients were categorized as having stage I disease and 52 (24.1%) as having stage II disease. Using the multivariate Cox model, age (hazard ratio [HR] 1.08, *p*<0.001), lymphatic invasion (HR 3.12, *p*=0.004), stage (HR 5.10, *p*<0.001), and CLR (HR 1.01, *p*=0.003) were significant determinants of OS. In addition, age (HR 1.11, *p*=0.002), lymphatic invasion (HR 3.16, *p*=0.010), stage (HR 6.89, *p*<0.001), and CLR (HR 1.05, *p*=0.002) were significant determinants of CSS.

**Conclusions:** Our findings show that CLR could be a determinant of survival in NSCLC patients undergoing curative surgical resection.

## Introduction

In patients with stage I or II Non-small cell lung cancer (NSCLC), surgical resection is widely accepted as the optimal therapeutic option 1. However, despite substantial advances in surgical techniques, adjuvant treatment (such as chemotherapy, epidermal growth factor receptor tyrosine kinase inhibitor, radiation therapy, or chemoradiation), and the development of new prognostic models, the prognosis remains far from satisfactory 2. Therefore, the search for crucial prognostic factors that can assist thoracic surgeons in identifying high-risk patients who may benefit from perioperative treatment or clinical trials is required.

The demographic and clinical variables that have been considered important determinants of survival of NSCLC are as follows: age, sex, body mass index (BMI), performance status, smoking, reduced forced expiratory volume in one second (FEV1), histology, tumor-node-metastasis (TNM) stage, T stage, N stage, tumor size, pleural invasion, lymphatic invasion, vascular invasion, type of surgery, and R0 resection (i.e., a microscopically margin-negative resection) 2-5. In addition, the impact of nutritional variables such as sarcopenia on surgical outcomes has been reported in NSCLC 6.

Moreover, the hematological and biochemical markers that have been reported in patients with NSCLC are as follows: C-reactive protein (CRP), peripheral blood lymphocyte count, the Glasgow prognostic score, neutrophil-to-lymphocyte ratio, platelet-to-lymphocyte ratio, lymphocyte-to-monocyte ratio, and monocyte-to-lymphocyte ratio 3, 7-9.

Recently, the clinical significance of the combination of peripheral blood lymphocyte count and CRP as prognosticators for gastrointestinal cancers (including gastric, colorectal, and pancreatic cancers) has been studied by some researchers 10-15. In a study by Kono et al., the CRP to lymphocyte ratio (CLR) was a determinant of the overall survival (OS) and disease-specific survival (DSS) in patients with gastric cancer 13. In addition, CLR is a feasible determinant of OS in pancreatic cancer 14. However, the clinical value of CLR has not been evaluated in tumors outside the gastrointestinal tract. Therefore, in this study, we assessed the clinical value of CLR in patients with NSCLC undergoing curative surgical resection.

## Methods

### Patients

The medical records of consecutive patients with NSCLC undergoing surgical resection from July 2006 to December 2019 at the Kyung Hee University Hospital at Gangdong were reviewed. The chest-abdomen-pelvis computed tomography (CT) scans and the combination of CT with positron emission tomography was a regular part of standard cancer staging. In addition, CT and magnetic resonance imaging were the main imaging techniques used to diagnose brain metastases.

The inclusion criteria were as follows: (i) primary NSCLC according to the 2015 World Health Organization classification of lung tumors 16; (ii) stage I or II, according to the 8th edition of the TNM classification (Union for International Cancer Control) 17; (iii) underwent pneumonectomy, sleeve lobectomy, bilobectomy, lobectomy, or segmentectomy; and (iv) underwent R0 resection. The exclusion criteria were: (i) underwent preoperative chemotherapy, radiotherapy, or another anti-cancer treatment prior to surgery; (ii) presence of small cell lung cancer, sarcomatoid carcinoma, or neuroendocrine tumor; (iii) previous malignancies within the last 5 years, or concurrent second malignancies; (iv) history of liver cirrhosis, stage 4 or 5 chronic kidney disease, (v) stage III or IV disease, (vi) positive test for human immunodeficiency virus, severe infections within 4 weeks prior to surgery, or active autoimmune diseases that required systemic or immunosuppressive agents at the time of surgery.

### Demographic and clinical variables

The variables collected in this study were as follows: age at surgery, sex, smoking history, BMI, Eastern Cooperative Oncology Group scale of Performance Status, percent predicted FEV1, types of surgery, histology, size and extent of the primary tumor, lymph node involvement, TNM stage, pleural invasion, lymphatic invasion, vascular invasion, and residual disease. A BMI of less than 18.5 kg/m^2^ was considered underweight. Classification of pleural invasion was as follows: PL0, tumor within the subpleural lung parenchyma or superficial invasion into the pleural connective tissue; PL1, tumor invasion beyond the elastic layer; PL2, tumor invasion to the pleural surface; and PL3, tumor invasion into any component of the parietal pleura 18. The classification of residual disease was as follows: R0, resection for cure or complete remission; R1, microscopic residual tumor; and R2, macroscopic residual tumor 19.

The laboratory variables collected were as follows: white blood cell count, differential count, hemoglobin concentration, serum creatinine level, serum albumin level, and CRP level. The CLR was calculated as follows: [CRP level (mg/dL) × 100]/peripheral blood lymphocyte count (number/μL), as reported previously 13, 14. Blood test results were analyzed through tests performed within 7 days before surgery. If more than one test result was available, the test result closest to the date of the surgery was selected for further analysis. The diagnosis of anemia was based on a hemoglobin concentration below 13 g/dL in men and 12 g/dL in women.

### Statistical analyses

Summaries of the data are expressed as the median with interquartile range, or number with percent. OS was measured from the date of surgical resection to the date of death from any cause or last follow-up. Cancer-specific survival (CSS) was measured from the date of surgical resection to the date of death due to cancer in the absence of other causes of death. Individuals who died of causes other than cancer were treated as censored.

The Cox proportional hazards model was used to calculate hazard ratios, which were performed only on variables that met the proportional hazards assumption on the basis of graphic plots of Schoenfeld residuals. Only variables with *p* <0.05 in the univariate analysis were included in the multivariate Cox model. Harrell's concordance probability (C-index) for the Cox model was performed to measure the discriminative power of a risk prediction model. The variance inflation factor (VIF) was calculated for the diagnosis of multicollinearity.

The decision curve analysis (DCA) displays estimates of the (standardized) net benefit over a range of probability thresholds used to categorize observations as 'high risk'. Bootstrap analysis with 500 resamples was used for the analysis.

In addition, a bootstrap cross-validation estimate of the C-index at different time points was applied to assess and compare the discriminative power of two regression models (i.e., baseline vs. full model). The number of bootstrap samples was 1000, and sampling with replacement from the original data set was accomplished.

The Mann-Whitney U test was used to compare differences between two independent groups when the dependent variable was continuous. All *p*-values presented were 2-sided, and statistical significance was declared at *p<*0.05. Data were analyzed using the R package.

## Results

### Demographic and clinical characteristics of the patients

A total of 331 patients were initially enrolled, and 117 patients were excluded for the following reasons: (i) 19 patients had concurrent malignant tumors or had other types of malignant tumors within 5 years; (ii) 4 patients had been diagnosed with neuroendocrine tumors, and 4 patients with sarcomas; (iii) 7 patients had been treated with chemotherapy or radiotherapy for NSCLC before surgery; (iv) 56 patients had stage III or stage IV disease; (v) 2 patients had no R0 resection; (vi) 9 patients had no available CRP data; (vii) 16 patients had undergone pulmonary metastasectomy for tumors other than lung cancer; and (viii) 4 patients had benign tumors of the lung. Finally, 214 patients with NSCLC were analyzed.

The most common surgical procedure was lobectomy, which was performed on 197 patients (77.3%), followed by segmentectomy (21.3%), and pneumonectomy (3%). The most common histological subtype was adenocarcinoma (69.9%), followed by squamous cell carcinoma (26.4%), adenosquamous cell carcinoma (1.4%), pleomorphic carcinoma (1.4%), and large cell carcinoma (0.9%). A total of 164 (75.9%) patients were categorized as having stage I disease, and 52 (24.1%) as stage II (Table 1).

### Determinants of survival

The median follow-up period was 34 months (range, 1-151 months). Using the univariate Cox model, age, sex, smoker, tumor size, nodal invasion, TNM stage, lymphatic invasion, vascular invasion, albumin level, and CLR were identified as significant determinants of OS. Using the multivariate Cox model, age (HR 1.08, 95% CI 1.04-1.14, *p*<0.001), lymphatic invasion (HR 3.12, 95% CI 1.43-6.81, *p*=0.004), stage (HR 5.10, 95% CI 2.45-10.59, *p*<0.001), and CLR (HR 1.01, 95% CI 1.00-1.02, *p*=0.003) were identified as significant determinants of OS. Harrell's C-index for the Cox model was 0.8337, indicating excellent discrimination. VIFs for age, lymphatic invasion, stage, and CLR were 1.10, 1.02, 1.01, and 1.19, respectively, indicating no significant collinearity between the covariates (Table 2).

Using the univariate Cox model, the same variables as those of OS were identified as significant determinants of CSS. Using multivariate analysis, age (HR 1.10, 95% CI 1.04-1.16, *p*=0.002), lymphatic invasion (HR 6.89, 95% CI 2.84-16.69, *p*<0.001), stage (HR 3.16, 95% CI 1.31-7.60, *p*=0.010), and CLR (HR 1.05, 95% CI 1.02-1.07, *p*=0.002) were identified as determinants of CSS. Harrell's C-index for the Cox model was 0.8571, indicating excellent discrimination. VIFs for age, lymphatic invasion, stage, and CLR were 1.10, 1.02, 1.01, and 1.10, respectively, indicating no significant collinearity between the covariates (Table 2).

**Figure 1:**
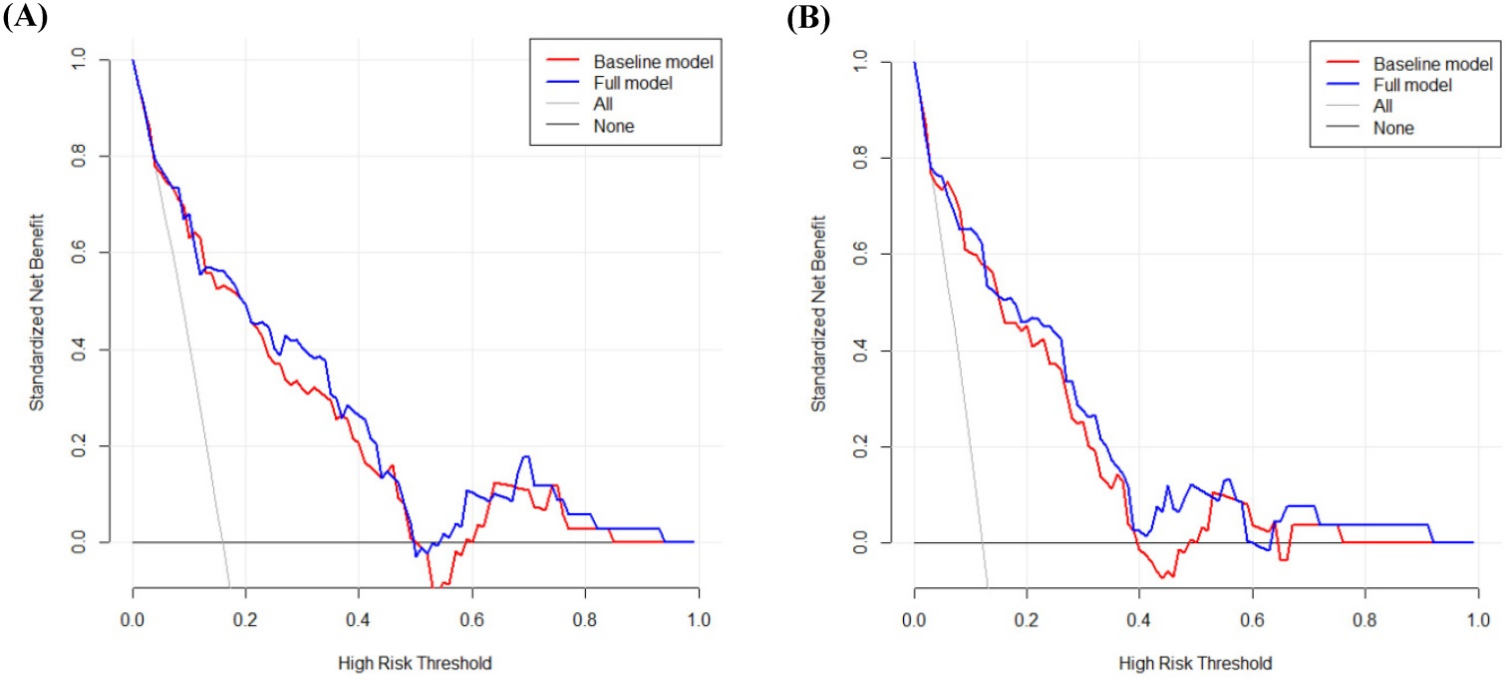
Decision curve analysis to calculate the clinical net benefit of each model for overall survival (A) and cancer-specific survival (B). The baseline model includes three covariates (i.e., age, lymphatic invasion, and stage), and the full model includes four covariates (i.e., age, lymphatic invasion, stage, and CLR). All, a line that all patients are surviving; None, a line that no patient is surviving.

**Figure 2:**
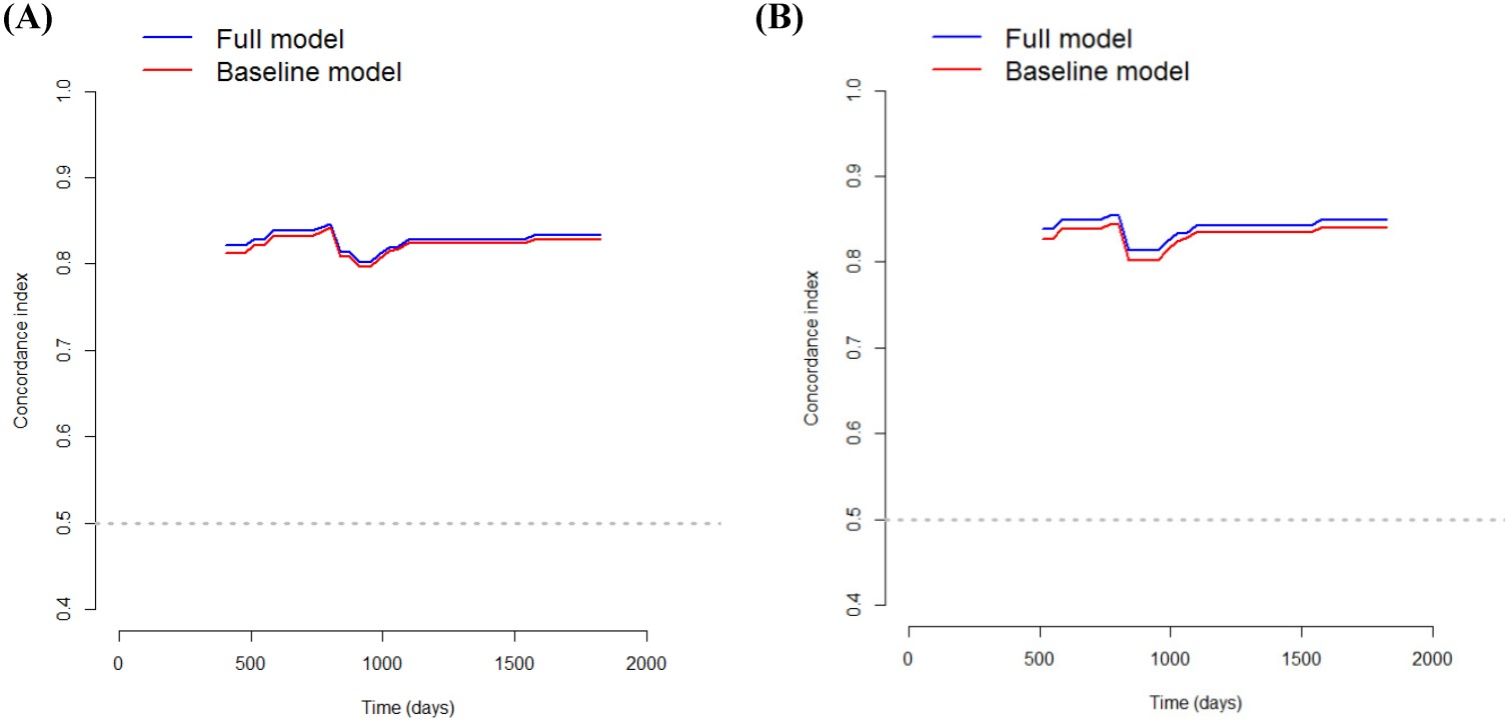
The bootstrap cross-validation estimates of the C-index at different time points to assess and compare the discriminative power of two regression models for overall survival (A) and cancer-specific survival (B). The baseline model includes three covariates (i.e., age, lymphatic invasion, and stage), and the full model includes four covariates (i.e., age, lymphatic invasion, stage, and CLR).

DCA was performed to calculate the clinical net benefit of the baseline model (i.e., three covariates of age, lymphatic invasion, and stage) and full model (i.e., four covariates of age, lymphatic invasion, stage, and CLR) at a certain range of threshold probability in predicting OS and CSS. The DCA revealed that the net benefit of the full model was superior to that of the baseline model at most of the threshold probabilities in terms of OS and CSS (Fig. 1A and Fig. 1B). In addition, bootstrap cross-validation also revealed higher C-indices for the full model (i.e., age, lymphatic invasion, stage, and CLR) than the baseline model (i.e., age, lymphatic invasion, and stage) in terms of OS and CSS (Fig. 2A and Fig. 2B).

### Correlation between basal patient characteristics and CLR

There were significant differences in CLR according to age (*p*=0.003), sex (*p*<0.001), smoking history (*p*<0.001), BMI (*p*=0.020), histology (*p*<0.001), tumor size (*p*<0.001), TNM stage (*p*=0.006), serum albumin level (*p*=0.001), and anemia (*p*<0.001) when Mann-Whitney U test was applied (Table 3).

**Figure 3:**
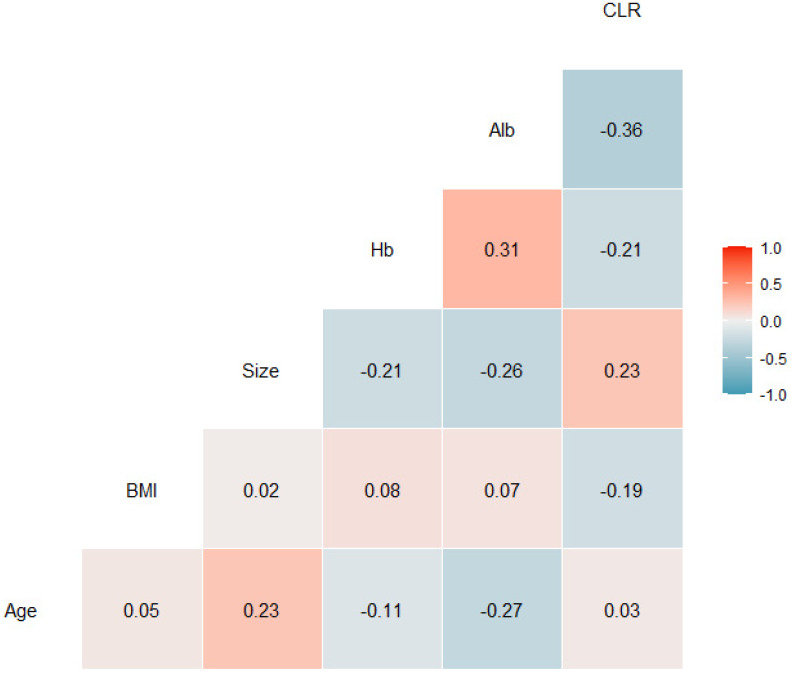
Correlation coefficients between patient characteristic and C-reactive protein to lymphocyte ratios. Alb, serum albumin level; BMI, body mass index; CLR, C-reactive protein to lymphocyte ratio; Hb, hemoglobin concentration.

In addition, there was a significant correlation between CLR and serum albumin level (*r*=-0.36). However, there were only weak correlations between CLR and age (*r*=0.03), BMI (*r*=-0.19), tumor size (*r*=0.23), and hemoglobin concentration (*r*=-0.21) using tests for Pearson correlation (Fig. 3).

## Discussion

The purpose of the present study was to assess the clinical feasibility of CLR in a cohort of NSCLC patients undergoing curative surgical resection. In this study, we found that preoperative CLR was a significant determinant of OS and CSS.

As CLR is a relatively new concept, there are only a few available studies, irrespective of cancer type. In a study by Kono et al., using a multivariate Cox model, CLR was found to be a significant determinant of OS and DSS in patients with gastric cancer undergoing curative surgery 13. In addition, in a study by Fan et al., pre-treatment CLR was found to be a feasible biomarker for the prediction of OS in patients with pancreatic cancer 14.

In our study, using the multivariate Cox model, CLR, along with three covariates (such as age, lymphatic invasion, and stages), was a significant determinant of both OS and CSS. Regarding OS, Harrell's C-index for the Cox model was 0.8337, indicating excellent discrimination. Moreover, the VIFs for age, lymphatic invasion, stage, and CLR showed no significant collinearity between these covariates. As for CSS, Harrell's C-index for the Cox model was 0.8571, indicating excellent discrimination. Furthermore, the VIFs for age, lymphatic invasion, stage, and CLR showed no significant collinearity between these covariates.

When excluding CLR from the model, the C-index for OS decreased to 0.8269, and that for CSS decreased to 0.8345, highlighting the significance of CLR as a determinant of survival. Bootstrap cross-validation also revealed a higher C-index for both OS and CSS in the full model (i.e., age, lymphatic invasion, stage, and CLR) than in the baseline model (i.e., age, lymphatic invasion, and stage), therefore, the full model including CLR seems to have wider clinical applicability than the baseline model (Fig. 2A and Fig. 2B). In addition, when CLR was replaced with CRP, the C-index for OS decreased to 0.8336, and that for CSS decreased to 0.8440. As such, the models including CLR rather than CRP are more predictive.

However, the mechanisms underlying the clinical significance of CLR remain unclear. CRP, an acute-phase reactant, is one of the most frequently used markers that reflects the body's systemic inflammatory response 20, 21. In patients with malignant tumors, CRP levels are reportedly modulated by cytokines, particularly interleukin 6, which is produced by the tumor cells themselves or by the surrounding cells 22. The role of CRP in tumorigenesis has also been elucidated in malignant tumors 23. Furthermore, in NSCLC, preoperative CRP has been suggested as a significant determinant of oncological outcome 3, 5, 24, 25. The peripheral blood absolute lymphocyte count (ALC) has been suggested as an important nutritional index in patients with various diseases 9, 26. In addition, its role as a marker for host cell-mediated cytotoxic immunity against infection and tumors has also been suggested 27. In NSCLC, preoperative ALC has been considered a good indicator of oncological outcome 9, 28, 29. As CRP reflects the level of the systemic inflammatory response 21 and ALC reflects the level of the nutritional and immunologic status 30, CLR could reflect the balance between inflammation, immunity, and nutrition in the body 10-15. In this study, relative increases in CLR levels were demonstrated in the elderly, male gender, former/current smoker, underweight, squamous cell carcinoma, increased tumor size, stage II, hypoalbuminemia, and anemia (Table 3). These findings may, at least in part, give an answer to why the CLR is an independent prognostic factor for OS and CSS in our study.

In the present study, age was associated with OS and CSS in a multivariate Cox model. The prognostic significance of age has been reported in previous studies on patients with NSCLC undergoing curative surgical resection 2-4, 7, 31, 32. In addition, lymphatic invasion was associated with poor OS and CSS rates in the multivariate Cox model. The prognostic significance of lymphatic invasion has been reported in previous studies on patients with NSCLC undergoing curative surgical resection 2, 8, 31.

The strengths of this study were as follows. First, the present study was the first to demonstrate the role of preoperative CLR as a determinant of survival in patients with NSCLC undergoing surgical resection. Using a multivariate Cox model, we found that CLR was a significant determinant of long-term outcomes (i.e., OS and CSS) along with age, lymphatic invasion, and stage. In this study, CLR was treated as a continuous variable, instead of being dichotomized according to the cutoffs. Optimizing cutoffs by minimizing the *p*-value can lead to bias, such as an overestimation of the prognostic impact, since cutoffs cannot be applied in different cohorts. In addition, dichotomization based on cutoffs falsely suggests that there are two qualitatively different subgroups when the prognostic impact is in fact linear 33. As such, our findings may help thoracic surgeons better differentiate patients with poor long-term survival using CLR with traditional stratification tools prior to therapeutic resection for NSCLC. Second, CLR is a combination of test items that are routinely tested before surgery, and it has the advantage of being able to obtain the test results quickly without the need for expensive test equipment.

This study had some limitations, therefore, the research results must be interpreted carefully. First, this study was conducted retrospectively. Therefore, missing data was inevitable and may have affected the results. Second, while both potential biases and random errors were controlled from the study plan to the implementation, our study had a limitation of single-center data analysis without validation through independent cohorts. However, based on the results of this study, it is possible to conduct a prospective study with an independent external verification group as the next step of this study.

In conclusion, our study demonstrated that preoperative CLR is an important determinant of OS and CSS in stage I and II NSCLC patients undergoing curative resection. However, external validation of our results is a prerequisite before they are subjected to clinical applications.

## Figures and Tables

**Table 1:** Demographic and clinical characteristics

Variables	Median (IQR), or *n* (%)
**Age, years**	69.0 (62.0-75.0)
**Sex**	
Male	136 (63.6)
Female	78 (36.4)
**Smoker**	
Current	42 (19.6)
Former	86 (40.2)
Never	86 (40.2)
**ECOG PS**	
0/1	206 (96.3)
2	8 (3.7)
**Percent predicted FEV1**	102.3 (90.0-115.0)
**BMI, kg/m^2^**	23.7 (21.8-25.8)
**Types of surgery**	
Pneumonectomy/lobectomy	168 (78.5)
Segmentectomy	46 (21.5)
**Histology**	
Adenocarcinoma	151 (70.6)
Squamous cell carcinoma	55 (25.7)
Others†	8 (3.7)
**Size of tumor, cm**	2.7 (1.9-3.5)
**T-stage**	
pT1	100 (46.8)
pT2	97 (45.3)
pT3	17 (7.9)
**N-stage**	
pN0	197 (92.1)
pN1	17 (7.9)
**TNM Stage**‡	
IA/IB	163 (76.2)
IIA/IIB	51 (23.8)
**Pleural invasion**	
PL0	157 (73.4)
PL1/2/3	57 (26.6)
**Lymphatic invasion**	
No	193 (90.2)
Yes	21 (9.8)
**Vascular invasion**	
No	204 (95.3)
Yes	10 (4.7)
**Albumin, g/dL**	4.1 (3.9-4.3)
**Anemia**§	
No	143 (66.8)
Yes	71 (33.2)
**CLR**	0.8 (0.4-2.6)

† Adenosquamous cell carcinoma (3 cases), pleomorphic carcinoma (3 cases), and large cell carcinoma (2 cases). ‡ AJCC 8th edition. § The cutoff point is 12 g/dL for female patients and 13 g/dL for male patients. IQR, interquartile range; ECOG PS, Eastern Cooperative Oncology Group performance status; FEV1, forced expiratory volume in one second; BMI, body mass index; PL, pleural invasion; TNM, tumor-node-metastasis; CLR, CRP to lymphocyte ratio.

**Table 2:** Univariate and multivariate Cox proportional hazards regression analysis of overall survival and cancer-specific survival

Covariates	Overall Survival		Cancer-specific Survival	
	HR (95% CI)	*p*-value	HR (95% CI)	*p*-value
**(A) Univariate analysis**				
Age, years†	1.09 (1.04-1.15)	<0.001	1.10 (1.04-1.17)	<0.001
Sex (female vs. male)	0.33 (0.14-0.79)	0.013	0.29 (0.10-0.85)	0.024
Smoker (current/former vs. never)	2.23 (1.05-4.74)	0.038	2.52 (1.01-6.30)	0.048
ECOG PS (2 vs. 0/1)	2.13 (0.51-8.93)	0.300	0.79 (0.34-1.81)	0.574
Underweight (yes vs. no)	1.95 (0.68-5.52)	0.212	1.47 (0.35-6.24)	0.601
Resection (Segmentectomy vs. PN/L)	0.62 (0.22-1.78)	0.374	0.56 (0.17-1.91)	0.359
Histology (SQC vs. non-SQC)	1.51 (0.99-2.29)	0.053	1.32 (0.81-2.13)	0.261
Size of tumor, cm†	1.68 (1.37-2.05)	<0.001	1.62 (1.30-2.23)	<0.001
Nodal invasion (yes vs. no)	2.41 (1.00-5.80)	0.050	3.64 (1.45-9.09)	0.006
TNM stage (II vs. I)	6.64 (3.35-13.16)	<0.001	8.77 (3.80-20.22)	<0.001
Pleural invasion (PL0 vs. PL1/2/3)	0.90 (0.41-1.99)	0.794	0.91 (0.37-2.27)	0.844
Lymphatic invasion (yes vs. no)	5.15 (2.45-10.87)	<0.001	5.71 (2.44-13.47)	<0.001
Vascular invasion (yes vs. no)	4.25 (1.48-12.17)	0.007	4.12 (1.22-13.93)	0.023
Albumin, g/dL†	0.20 (0.08-0.48)	<0.001	0.24 (0.09-0.70)	0.009
Anemia (yes vs. no) ‡	1.92 (0.99-3.69)	0.052	1.71 (0.79-3.72)	0.173
CLR†	1.03 (1.01-1.06)	0.004	1.04 (1.01-1.06)	0.002
**(B) Multivariate analysis**				
Age, years†	1.09 (1.04-1.15)	<0.001	1.10 (1.04-1.16)	0.002
TNM stage (II vs. I)	5.10 (2.45-10.59)	<0.001	6.89 (2.84-16.69)	<0.001
Lymphatic invasion (yes vs. no)	3.15 (1.45-6.86)	0.004	3.16 (1.31-7.60)	0.010
CLR†	1.04 (1.02-1.07)	0.001	1.05 (1.02-1.07)	0.002

† Continuous variables. ‡ The cutoff point is 12 g/dL in female patients and 13 g/dL in male patients. HR, hazard ratio; CI, confidence interval; ECOG, Eastern Cooperative Oncology Group; PFP, percent FEV1 predicted; PN/L, pneumonectomy or lobectomy; PL0, tumor within the subpleural lung parenchyma or invading superficially into the pleural connective tissue; PL1, tumor invaded beyond the elastic layer; PL2, tumor invaded the pleural surface; PL3, tumor invaded any component of the parietal pleura; TNM, tumor-node-metastasis; SQC, squamous cell; CLR, CRP to lymphocyte ratio.

**Table 3:** C-reactive protein to lymphocyte ratios according to the covariates

Covariates	CLR	
	Median (IQR)	*p*-value
**Age, years**		
<65	0.51 (0.23-1.76)	0.003
≥65	1.00 (0.51-3.00)	
**Sex**		
Male	1.19 (0.47-4.31)	<0.001
Female	0.57 (0.25-1.20)	
**Smoking**		
Never	0.57 (0.31-1.19)	<0.001
Former/current	1.38 (0.47-4.46)	
**ECOG PS**		
0/1	0.81 (0.43-2.57)	0.356
2	1.55 (0.97-6.15)	
**BMI, kg/m^2^**		
<18.5	2.41 (0.96-15.71)	0.020
≥18.5	0.81 (0.42-2.37)	
**Histology**		
SQC	2.93 (1.22-8.14)	<0.001
Non-SQC	0.62 (0.35-1.54)	
**Size of tumor, cm**†		
<4.0	0.72 (0.41-1.82)	<0.001
≥4.0	2.70 (0.81-5.65)	
**Nodal invasion**		
No	0.87 (0.44-2.93)	0.129
Yes	0.53 (0.30-1.13)	
**TNM stage**‡		
IA/IB	0.73 (0.41-1.81)	0.006
IIA/IIB	1.78 (0.55-5.11)	
**Pleural invasion**		
PL0	0.82 (0.43-2.46)	0.639
PL1/2/3	0.87 (0.43-2.46)	
**Lymphatic invasion**		
No	0.85 (0.43-2.48)	0.657
Yes	0.81 (0.30-2.93)	
**Vascular invasion**		
No	0.83 (0.43-2.51)	0.570
Yes	1.26 (0.54-4.78)	
**Albumin, g/dL**		
<3.5	9.90 (3.24-18.28)	0.001
≥3.5	0.81 (0.42-2.26)	
**Anemia**§		
No	0.73 (0.39-1.61)	<0.001
Yes	1.71 (0.58-6.75)	

† Determined by a receiver operating characteristic curve analysis. ‡ AJCC 8^th^ edition. § The cutoff point is 12 g/dL in female patients and 13 g/dL in male patients. CLR, C-reactive protein to lymphocyte ratio; IQR, interquartile range; ECOG PS, Eastern Cooperative Oncology Group performance status; BMI, body mass index; SQC, squamous cell; TNM, tumor-node-metastasis; PL, pleural invasion.
